# The Role of Dopamine and Glutamate Modulation in Huntington Disease

**DOI:** 10.3233/BEN-2012-120268

**Published:** 2013

**Authors:** Sumeer K. Mittal, Clare Eddy

**Affiliations:** The Michael Trimble Neuropsychiatry Research GroupDepartment of NeuropsychiatryBSMHFT and University of BirminghamBirminghamUK

**Keywords:** Anti-dopaminergic, anti-glutamatergic, dopamine, glutamate, Huntington's disease, pharmacotherapy, treatment

## Abstract

*Background:* Huntington disease (HD) is an inherited neuropsychiatric condition with progressive neurodegenerative changes, mainly affecting the striatum. Pathological processes within the striatum are likely to lead to alterations in dopamine and glutamate activity in frontostriatal circuitry, resulting in characteristic motor, behavioural and cognitive symptoms.

*Methods:* We conducted a systematic literature search in order to identify and review randomised, double-blinded, placebo-controlled trials of anti-dopaminergic and anti-glutamatergic therapy in HD.

*Results:* Ten studies satisfied our selection criteria. These studies investigated a range of agents which act to antagonise dopamine (tetrabenazine, typical and atypical antipsychotics) or glutamate (amantadine, riluzole) transmission.

*Discussion:* Although most agents showed efficacy in terms of amelioration of chorea, the available evidence did not allow us to identify a universally effective treatment. One difficulty associated with analysing the available evidence was a high prevalence of side effects, which prevented the full therapeutic potential of the medications from being adequately investigated. A further limitation is that many studies evaluated treatment effectiveness only in relation to patients' motor symptoms, even though behavioural and cognitive changes may negatively impact patients' quality of life. There is a clear need for further higher-level evidence addressing the effects of dopaminergic and glutamatergic agents on global functioning in HD.

## 1. Introduction

Huntington disease (HD) is a hereditary, hyperkinetic movement disorder, with a prevalence of approximately 4–8/100,000 in most countries. HD involves progressive neurodegenerative changes primarily affecting GABAergic striatal intereurons. The autosomal dominant mutation is of the huntingtin gene, located on the short arm of the 4$^{th}$ chromosome. This gene contains a repeated section of the trinucleotide CAG [1]. An enlargement of the CAG repeat sequence results in the patient expressing the characteristic features of the Huntington phenotype. The severity of a patient's symptoms is dependent on the length of the CAG repeats [1,2].

In addition to characteristic motor symptoms such as chorea, patients with HD often present with behavioural and cognitive symptoms. These include psychiatric and mood changes such as depression and apathy, and behavioural changes including irritability and aggression [3]. Cognitive difficulties include deficits in executive functions such as working memory and response inhibition [4], with dementia becoming more apparent with disease progression.

Converging lines of evidence indicate that excessive activity within dopamine and glutamate pathways can affect inhibition and excitation processes within the basal ganglia, contributing to the symptoms of HD (Table 1) [1,2,5]. For example, the neurodegenerative processes seen in HD could be linked to a relative excess of neurotransmitters such as glutamate [5], which can contribute to cell death through excitotoxicity. It has also been suggested that there is an excess of dopamine production from the substantia nigra-pars compacta (SNc) in HD [2,5]. This could lead to excessive of striatal D2

ISSN 0953-4180/13/$27.50 © 2013 – IOS Press and the authors. All rights reserved

Table 1
Summary of main symptoms experienced in patients with Huntington disease

| Motor symptoms | Behavioural symptoms | Cognitive impairment |
|---|---|---|
| Stereotypical involuntary choreiform movements (over time chorea disappears and patient develops dystonia and rigidity). | Psychosis | Dementia |
| Problems with normal voluntary movements such as speech, swallowing, gait and eye movements. | Irritability | Difficulty in multitasking and planning ahead. |
| | Aggression | Impairment of executive function, delay in acquisition of new skills. |
| | Frustration | |
| | Depression | |

receptors, reducing the ability to suppress movement via the indirect pathway through the basal ganglia. In addition, increased inhibition of basal ganglia nuclei involved in the indirect pathway leads to decreased inhibition of the ventrolateral (VL) thalamus, which in turn results in hyper-excitability of the motor cortex. Dysfunction within the direct and indirect pathways leads to involuntary choreiform movements.

Pharmacotherapy in HD aims to target specific neurochemical alterations within frontostriatal circuitry, in order to reduce chorea and other symptoms related to HD. Agents which have been trialled include anti-dopaminergic and anti-glutamatergic drugs which serve to reduce dopamine and glutamate levels. In this paper we review the efficacy of tetrabenazine (a dopamine depletor), a range of neuroleptics (dopamine antagonists) and anti-glutamatergic therapy (e.g. amantadine) using high level evidence from clinical trials. These studies indicate that a number of agents may be effective in treating different aspects of HD, although they are not without associated drawbacks.

## 2. Method

Our search was carried out in line with the Prisma guidelines [6]. The following databases were searched between 1950 and May 2011: Medline, PubMed and PsycINFO. The search terms used were "Huntington", "treatment", "pharmacotherapy", "tetrabenazine", "neuroleptic" and "glutamat*". Duplicates were removed and the remaining articles were then searched manually. Only double-blind, randomised trials were included. Articles which did not involve human participants or were not written in English were also excluded. To supplement these papers, a grey literature search was carried out. The reference lists of all relevant articles that satisfied the criteria of the manual search were reviewed to identify further relevant studies. After the selection of relevant articles, it became apparent that the studies were published in similar journals. These included Movement Disorders, Neurology, Clinical Neuropharmacology and Journal of Neurology, Neurosurgery and Psychiatry. The next phase of the search process was to use the same criteria that were used in the database searches with the search engines of these respective journals. Finally, the Google engine was also employed to search for further literature that may have not have been identified through the database searches.

## 3. Results

The results of the literature search are shown in Tables 2–5. Ten studies were identified which satisfied the search criteria and aims of this review (3 trials of tetrabenazine, 3 of neuroleptics and 4 of anti-glutamatergics). The trials were conducted between the years of 1979 and 2008. They varied greatly in their sample size, ranging from 1–537 subjects. The length of most of the trials was between a few hours and 10 weeks, although one study lasted for 3 years. There was also a high degree of variation in the dose of the trial drug administered, 100 mg being the lowest and 1200 mg being the highest. There was however some consistency across trials in terms of the assessment tools used to determine efficacy: the Unified Huntington's Disease Rating Scale (UHDRS) [7] being the most common. Studies which pre-dated the introduction of the UHDRS tended to apply more subjective measures.

## 4. Discussion

### *4.1. Anti-dopaminergic therapy*

#### *4.1.1. Tetrabenazine*

Tetrabenazine (TBZ) is a catecholamine depleting agent. Although initially developed for the treatment of

Table 2
Results of the systematic literature search on anti-dopaminergic and anti-glutamatergic pharmacotherapy for Huntington disease

| Study and country | Drug therapy | Control agent | Dose* | n | Length of study | Assessment tool(s)* | % with moderate-severe side effects in treatment arm** | Trial effect |
|---|---|---|---|---|---|---|---|---|
| HSG (2006)[14] United States | Tetrabenazine | Placebo | Variable (max 100 mg/day) | 84 | 7 weeks | UHDRS, CGI, UPDRS, Barnes Akathisia Scale | 68.5 | P |
| Frank et al. (2008) [15] United States | Tetrabenazine | N/A – as it was a TBZ withdrawal trial | N/A – as it was a TBZ withdrawal trial | 30 | 5 days | UHDRS | 20.8 | N |
| Jankovic (1982) [8] United States | Tetrabenazine | Placebo | 200 mg/day | 1 | 10 weeks | Clinician assessment of hyperkinesia in addition to video analysis | 0.0 (trial with only 1 patient who had mild side effects) | P |
| Van Vugt et al. (1997) [19] Netherlands | Clozapine | Placebo | Variable (max 150mg/day) | 33 | 31 days | UHDRS, AIMS | 82.3 | N |
| Caine et al. (1979) [20] United States | Clozapine | Placebo | Variable (max 500mg/day) | 3 | 4 weeks | Clinician assessment | 33.3 | P |
| Quinn and Marsden. (1984) [14] United Kingdom | Sulpiride | Placebo | Variable (300 mg–1200 mg/day) Mean dose 1055 mg/day | 11 | 4 weeks | Chorea severity score and functional assessment by clinician | 18.2 | P |
| Lucetti et al. (2003) [21] Italy | Amantadine | Placebo | 200 mg (IV infusion) | 9 | 5 hours (300 min IV infusion) | UHDRS, AIMS | N/A (tolerated well by all 9 patients) | P |
| O'Suilleabhain and Dewey (2003) [22] United States | Amantadine | Placebo | 300 mg/day | 24 | 2 weeks | Chorea scale and video analysis | 41.7 | N |
| HSG (2003) [23] United States | Riluzole | Placebo | 100mg/day OR 200mg/day | 63 | 8 weeks | UHDRS | 48.8 | P |
| Landwehrmeyer et al. (2007) [24] 8 European Countries | Riluzole | Placebo | 100mg/day | 537 | 3 years | UHDRS | 21.8 | N |

Abbreviations: HSG = Huntington Study Group. UHDRS = Unified Huntington's Disease Rating Scale. AIMS = Abnormal Involuntary Movements Scale. CGI = Clinical Global Improvement. UPDRS = Unified Parkinson's Disease Rating Scale. "P = Positive results in favour of treatment." "N = Negative results not in favour of treatment."
*Further information regarding dosages and how the assessment took place can be found in Discussion section under each treatment therapy.
**Percentages were calculated from data on side effects in each article.

schizophrenia it has shown therapeutic potential in hyperkinetic movement disorders such as HD. One of the first randomised trials investigating TBZ, which was carried out by Jankovic in 1982 [8], showed that TBZ was quite effective against hyperkinesia in patients with a variety of different movement disorders. A patient with HD showed marked improvement in chorea after TBZ treatment. Side effects included insomnia, restlessness and anxiety. However, Jankovic concluded TBZ was a useful and safe treatment in at least some patients with hyperkinetic movement disorders.

TBZ selectively binds to the CNS vesicular monoamine transporter VMAT2 with high affinity, resulting in depletion of dopamine, noradrenaline and serotonin [9–11]. However, it is thought to be dopamine which is most sensitive to the effects of TBZ [12]. It is impor-

Table 3
Further information on dose and assessment regime in studies investigating TBZ

| Study | No. of subjects (subsequent to randomisation) | | Drug therapy | | Method of assessment |
|---|---|---|---|---|---|
| | Treatment arm | Placebo arm | Treatment arm | Placebo arm | |
| HSG (2006) [14] | 54 | 30 | – 12.5 mg tablet of TBZ given bd in week 1.<br>– Increased by 1 tablet every week (for 7 weeks) until desired anti-choreic effect achieved or intolerable side effects.<br>– Remained on same dose for 5 weeks afterwards | Identical oral placebo capsules | – Patients assessed at 1, 3, 5, 7, 9, 12 and 13 weeks using the UHDRS total maximal chorea score, the Clinical Global Impression Scale and Functional Impact Scale.<br>– Development of Parkinsonian like symptoms as side effects can arise, following long-term TBZ use. For this reason, the trial also measured speech, swallowing and akathisia using the UPDRS and Barnes Akathisia Scale. |
| Frank et al. (2008) [15] | N/A* | N/A* | N/A* | N/A* | – Primary outcome was to evaluate the level of chorea at day 3, comparing the W group with the PW and NW group combined who would still be on TBZ at day 3.<br>– UHDRS chorea score was implemented.<br>– Secondary outcome measure was the change in total functional capacity (TFC). |
| Jankovic (1982) [8] | 1 | 1 | 200 mg/day TBZ | Identical oral placebo capsules | – Own rating scale was used to assess hyperkinesia.<br>– Video clips taken of patients and analysed by independent neurologists. |

*The study by Frank et al. [11] was a TBZ withdrawal trial. There were three different groups, one that remained on TBZ, one that had TBZ withdrawn after 1 day and the other had TBZ withdrawn after 3 days.

tant to note that certain regions of the basal ganglia including the caudate, striatum and nucleus accumbens are most affected by HD. Part of the reason for the therapeutic effectiveness of this agent may therefore lie in the finding that these areas also have the highest binding capacity for TBZ [1,13].

In 2006 the Huntington Study Group (HSG) looked at TBZ efficacy [14] in terms of treating chorea as assessed using the UHDRS scale. The UHDRS was developed by the HSG, to ensure the uniform assessment of outcomes in clinical trials [7]. The HSG reported that by weeks 9–12, there was a mean reduction in chorea severity of 23.5% from the baseline measurements in the TBZ group. This was mean 5 UHDRS units, compared to a reduction of mean 1.5 UHDRS units in those on placebo. Assessment with the Clinical Global Improvement scale (CGI) also showed TBZ as favourable over placebo. In those receiving TBZ, 69% achieved an improvement in the CGI score of $\leqslant 3$ ($< 4 =$ improvement, $> 4 =$ worsening), compared with 24% of patients on placebo [14]. This significantly better outcome, of almost three times, is extremely supportive of the benefits of TBZ. Furthermore, the authors found a positive correlation between improvement in chorea and CGI score. These data supports the role of TBZ in the amelioration of chorea in HD patients. However, there were five study withdrawals in the TBZ group compared to only one in the placebo group, and some adverse events (including suicide/suicidal ideation and a fall) were noted.

An different approach was adopted in a study conducted by Frank et al. [15] in 2008. These authors analysed the efficacy of TBZ in reducing chorea, by confirming that the choreiform movements return when those who take TBZ in the long-term are withdrawn from their medication. Subjects were screened over a 13 month period and randomised into three parallel groups. The first group (12 subjects) was the withdrawal group (W), withdrawn from treatment with TBZ after 1 day. The second group (12 subjects) was the partial withdrawal group (PW), withdrawn after 3 days. The third group (6 subjects) was the non-withdrawal group

Table 4
Further information on dose and assessment regime in trials investigating neuroleptic therapy

| Study | No. of Subjects (subsequent to randomisation) | | Drug therapy | | Method of assessment |
|---|---|---|---|---|---|
| | Treatment arm | Placebo arm | Treatment arm | placebo arm | |
| Van Vugt et al. (1997) [19] | 7 + 4* | 11 + 4* | Initial dose of 25 mg/day clozapine. Increased in 25 mg increments every other day. | Identical oral placebo capsules | – AIMS and UHDRS chorea scores.<br>– Functional capacity assessment, using a 21 item self-questionnaire from which a total point score was determined and analysed by independent blinded adjudicators. |
| Caine et al. (1979) [20] | 3 | 3 | Three variable doses of 60, 200, 500 mg/day. | Identical oral placebo capsules | – Clinician assessment of hyperkinesia |
| Quinn et al. (1984)[16] | 11 | 11 | Initial dose 300 mg/day. Increased in 300 mg increments by the fourth week to a max of 1200 mg/day. | Identical oral placebo capsules | – Assessed at the end of weeks 2 and 4, using 3 criteria.<br>– Firstly, movement count was assessed using an easily identifiable repetitive movement, at a frequency easy to measure, such as lip pouting.<br>– Secondly, a 'chorea severity score'.<br>– Lastly, a functional score whereby a 'functional disability scale' was designed incorporating several symptoms. |

*Neuroleptic treated and naïve groups respectively.

(NW), and remained on TBZ treatment. Frank et al. showed that the mean UHDRS chorea score increased by 5.3 units in the W group from days 1 to 3, compared with an increase of only 3.0 units in the 'combined' PW and NW groups. These results provide some evidence in support of TBZ being effective in controlling chorea, and no adverse effects were reported after abrupt treatment withdrawal. However, there were some limitations associated with this study. These include that the randomisation process was not fully stratified, which lead to an uneven distribution of prognostic factors. There were also differences in mean baseline chorea scores and motor characteristics between the NW group and PW + W combined groups. For example, the NW group had a mean UHDRS motor score of 50.0 whereas the W and PW group had an average motor score of 31.4. Indeed, the authors commented on the fact that the NW group generally had more advanced disease. Such factors make it harder to make accurate comparisons between the different treatment groups.

#### *4.1.2. Anti-psychotics*

Neuroleptic drugs block the activity of dopamine due to antagonistic effects on dopamine D2 receptors [1,2, 5]. However, they may also help treat the psychiatric symptoms of HD (such as depression) through agonist effects on serotonin 5-$HT_{1A,2A}$ receptors [5]. Atypical neuroleptics in particular may show a greater affinity for serotonin receptors in comparison with dopamine D2 receptors [1,5]. An abundance of case reports, case-series and follow up studies have been conducted on the use of neuroleptics in the treatment of HD symptoms. Despite this, there is a paucity of high level evidence from randomised, controlled trials.

##### *4.1.2.1. Typical neuroleptics*

A trial carried out by Quinn and Marsden [16] in 1984 evaluated the effects of sulpiride in 11 HD patients and 9 patients with tardive dyskinesia. The 11 patients with HD had a mean age of 53 years (range 37–63) and had displayed motor symptoms of the disease for a mean of 6.7 years (range 3–16). Sulpiride was linked to a median movement count of 14 compared to 38 for the patients on placebo. A difference of 63% was attributed to the sulpiride and was statistically significant at the 0.01% level. Moreover, there was a significant difference of 4 points in the chorea severity score between the two groups. Despite these findings, sulpiride treatment was not found to lead to an improvement in the functional score.

Two other trials looking at another typical neuroleptic, the substituted benzamide tiapride, reported similar findings in that improvements in chorea were seen. However, when functional capacity scores were compared there was no significant difference between the treatment and placebo groups [17,18].

Table 5
Further information on dose and assessment regime in trials investigating anti-glutamatergic therapy

| Study | No. of Subjects (subsequent to randomisation) | | Drug therapy | | Method of assessment |
|---|---|---|---|---|---|
| | Treatment arm | Placebo arm | Treatment arm | Placebo arm | |
| Lucetti et al. (2003) [21] | 9 | 9 | 2 hour IV infusion of amantadine (200 mg dissolved in 500ml normal saline). | 2 hour IV infusion of 500 ml normal saline. | – A blinded neurologist scored the patient using the AIMS score (1–4) looking at the face, neck, trunk and limbs for hyperkinesia every 15 minutes.<br>– The motor component of UHDRS was also used.<br>– A second neurologist rated the patients using video clips taken for 60 seconds every 15 minutes. |
| O'Suilleabhain and Dewey (2003) [22] | 11 | 13 | 300 mg (100 mg tds) amantadine hydrochloride capsules. | Identical placebo capsules tds. | – 30 second video clips were taken of each patient at baseline and subsequent to the treatment. Reviewed by two independent physicians.<br>– The authors implemented their own chorea scale from 0 (absent chorea) to 4 (marked/prolonged chorea).<br>– Rated 6 different body parts: neck, trunk and every limb creating a maximum possible score of 24. |
| HSG. (2003) [23] | 18 + 23 | 22 | SPLIT INTO TWO.<br>-1. 100 mg riluzole/day<br>-2. 200 mg riluzole/day | Identical oral placebo capsules bd. | – Complete UHDRS assessment was taken at baseline and subsequent to treatment analysing motor, cognitive, behavioural and functional symptoms.<br>– Primary outcome was change in UHDRS chorea score and secondary outcomes focussed on all other aspects of the UHDRS. |
| Landwehrmeyer et al. (2007) [24] | 357 | 180 | 100mg riluzole (50mg bd) for 3 years. | Identical oral placebo capsules for 3 years. | – UHDRS motor score (MS) and UHDRS total functional capacity<br>– Both of these measurements considered as one entity giving a combined score (CS). |

*4.1.2.2. Atypical neuroleptics*

One benefit of the use of atypical neuroleptics is that they are thought to be associated with a much lower incidence of extra-pyramidal side effects than typical neuroleptics and may therefore prove to be better tolerated.

In 1997 Van Vugt et al. [19] investigated the effects of clozapine on 33 HD patients. Twelve patients were already taking neuroleptic medication before entering the trial. As a result, the authors decided to divide the population into two groups (previously neuroleptic treated and neuroleptic naïve). Randomisation to either placebo or clozapine took place within each of these categories, to remove the confounding factor of concomitant neuroleptic therapy.

For the neuroleptic naïve group, clozapine was responsible for a reduction in chorea by 7.3 on the Abnormal Involuntary Movements Scale (AIMS) and 4.0 units on the UHDRS, compared with 0 and 0.3 respectively for placebo. However, this difference was not statistically significant. In the neuroleptic treated group, clozapine had no effect on chorea compared to placebo. There was also no effect on voluntary movement. Independent investigators who analysed the video recordings reported no difference between clozapine and placebo, for either of the two groups. Furthermore, functional capacity assessment revealed that all patients on clozapine reported a reduction in functional capacity compared to those on placebo. This may be related to the finding that all patients randomised to clozapine reported having experienced side effects, which included drowsiness, dizziness, walking difficulties, fatigue and hypersalivation. In fact, the pre-defined maximum dose of 150mg/day of clozapine was only achieved in three patients. Although this was a well-designed trial in many respects, an inability to achieve the dose set out in the protocol means that the efficacy of clozapine in reducing chorea was not fully investigated.

Contrasting findings were reported in a trial conducted in 1979 by Caine et al. [20]. Clozapine was investigated in 12 patients with hyperkinetic movement disorders, three of whom were HD patients. The study reported 'marked' improvements in chorea. Two of the patients tolerated high doses of 200 and 500 mg/day respectively. The third patient failed to complete the trial due to slurred speech, drowsiness and orthostatic hypertension on a dose of 60 mg. This trial predates the UHDRS and other more objective measures of chorea. This study indicates that tolerance could be an issue with clozapine, although the study was limited by small sample size.

No randomised, controlled trials of atypical neuroleptics have been conducted since the publication of the article by Van Vugt et al. [19]. However, there are increasing numbers of case-reports and cohort studies looking at neuroleptic therapy in symptomatic treatment of HD. An important limitation of the aforementioned studies is that they neglected to consider the psychiatric and cognitive symptoms of HD patients, and instead focused exclusively on chorea. Further placebo-controlled studies, with larger sample sizes are needed to investigate the benefits of neuroleptic therapy on a wider spectrum of HD symptoms.

### 4.2. *Anti-glutamatergic therapy*

It has been suggested that excitotoxicity may play a role in HD as in other neurodegenerative conditions such as Parkinson's and Alzheimer's disease [2, 5]. Riluzole and amantadine are two anti-glutamatergic therapies that have been investigated in rigorous trials in HD.

Lucetti et al. [21] investigated the efficacy of intravenous and oral amantadine, a non-competitive antagonist of the NMDA receptor, in 9 HD patients in a randomized crossover trial. In the amantadine arm, there was a significant reduction in the average AIMS score from 20 at baseline to 9.5 '90 minutes after initiation of the infusion'. There was only a reduction of 2 units, from 20 to 18, in the placebo group. No significant differences in other motor symptoms according to the UHDRS scale were found between the two treatment arms. These results contrast with the findings of O'Suilleabhain and Dewey [22] who carried out a crossover trial involving 24 HD patients, as these authors reported no change in chorea score in the amantadine treated group from baseline. Despite this, 19 patients subjectively reported feeling an improvement with amantadine (e.g. improved balance, alertness, coordination) versus 6 with placebo, and this difference was significant. Furthermore, patients taking amantadine reported improved quality of life. Adverse effects were reported by 10 patients taking amantadine and included insomnia, agitation and anxiety.

There are at least two possible explanations for the conflicting findings of Lucetti et al. and O'Suilleabhain and Dewey. Firstly, there may be a difference depending on whether amantadine is delivered intravenously or orally. In Lucetti et al.'s study, 200 mg of amantadine dissolved in 500mg of saline was delivered over 2 hours. Moreover, its affects were analysed immediately in 15 minute intervals, from the moment of infusion. In the study carried out by O'Suilleabhain and Dewey, amantadine was delivered orally in three separate daily doses of 100 mg, over 2 weeks. When absorbed via the gastrointestinal tract, it may take longer for amantadine to enter the bloodstream. In the latter study, measurements of chorea were only recorded at the end of a two week period, at which point serum concentrations of amantadine are unlikely to be as high as in those patients who were administered IV infusions. Secondly, there was a discrepancy in the rating scales used in the two trials.

Riluzole is a drug which inhibits glutamate release and the current evoked by the stimulation of excitatory amino acid receptors [23]. The HSG looked at the effects of riluzole on HD patients in 2003 [23]. Compared to baseline figures, those on a riluzole dose of 100mg saw a mean reduction in chorea of 0.2 ($\pm$ 2.9) UHDRS units. Those on a higher riluzole dose saw a 2.2 unit reduction ($\pm$ 3.3). This was in comparison to a slight increase in chorea of 0.7 units ($\pm$ 3.4) with placebo [23]. However, although riluzole improved choreiform movements, changes in motor score or total functional capacity were not significant, and no improvement in cognitive or behavioural symptoms was seen. As with the previous therapies discussed, medication side effects were apparent. The higher dose riluzole group reported more side effects than the lower dose riluzole group, and both of these groups reported significantly more side effects versus placebo. Careful titration of dose may allow a balance to be struck between chorea amelioration and toxicity.

A much larger 3-year long trial investigated the effects of riluzole on 537 patients with HD [24]. One contrasting strength of this study compared to many other trials is that efficacy was assessed based on a wider range of aspects of HD than just motor symptoms. Patients were randomized to treatment or placebo (2:1 ratio). Only 379 of the original 537 participants completed the study (many participants were excluded because they required concomitant medication for the treatment of chorea). The results were almost identical between the placebo and treatment group. There was no difference for treatment versus placebo. Riluzole therefore appeared to have no neuroprotective or beneficial symptomatic effects. The strengths of this study include investigation of a large sample size of HD patients from 44 centres across 8 European countries, over a long period of time. As a result, the findings are very generalizable.

## 5. Conclusions

It seems that the two agents which demonstrated the greatest overall improvements in chorea were sulpiride in the study carried out by Quinn et al. [16] and TBZ in the trial conducted by in the HSG [14]. Both of these studies reported significant changes in chorea from baseline. However, only the TBZ study investigated and reported benefits of therapy on functional capacity, showing TBZ to be effective in improving functional ability, with significant differences observed on the CGI scale. Having said this, TBZ was associated with side effects such as insomnia and anxiety, which may make it difficult to tolerate in some cases. There is also the possibility that TBZ may reduce serotonin levels, which could affect mood. In contrast, some neuroleptics such as sulpiride could act as as serotonergic agonists, which could make them most beneficial in cases where depression is seen. The atypical neuroleptic clozapine is unlikely to improve chorea and can be associated with toxicity issues. In relation to glutamatergic agents, the evidence for improved chorea with amantadine is mixed, although there is some evidence it may help with other factors such as coordination and alertness, and importantly, improve quality of life. Riluzole may help reduce chorea but perhaps not functional capacity, but high doses can lead to intolerable side effects.

There are a number of limitations associated with this review. Firstly, we were only able to identify a relatively small number of studies which satisfied our inclusion criteria. Although this is likely to reflect the scarcity of higher level evidence, there may have been some useful studies which were not included. Secondly, it was difficult to draw comparisons across studies. In many cases this is due to differences in methodology (e.g. dosages, differences in assessment tools etc.). Thirdly, the studies were unable to demonstrate the efficacy of the therapies in reducing cognitive or behavioural/psychiatric symptoms. The main reason for this is that the main focus in the reviewed trials was chorea. However, another limitation could be the occurrence of adverse side effects. The ability to fully assess psychiatric symptoms would have been made difficult by the emergence of new similar symptoms as side effects. Furthermore, the presence of side effects makes it very difficult to determine a single best treatment and highlights the importance of including quality of life measures.

There are key factors which should be taken into account when developing future trials. The authors of the trials included in this review all commented on the lack of randomised controlled trials. However, of further necessity is assessment involving larger sample sizes, longer time periods and assessment of the effects of a range of doses and methods of administration of different agents. Comparisons of patient groups with different characteristics (e.g. different ages, CAG repeat lengths etc.) will allow clinicians to make more specific treatment recommendations tailored to the individual. Critically, some studies [25] indicate that movement symptoms such as chorea are not always closely related to patients' reports of personal wellbeing. Thorough assessment of therapeutic potential should therefore encompass treatment effects on movement symptoms, cognitive and psychiatric aspects of HD, side effects, and outcome in terms of both functional capacity and perceived quality of life.

Finally, It is important to note that pharmaceutical intervention is not the only avenue that is being explored in the fight against HD. Research is being carried out into 'gene therapy' and methods that could possibly 'switch off' the huntingtin gene (or the mutated part alone). The combination of pharmacotherapy with novel approaches is likely to be crucial in the multi-disciplinary management of HD.
